# Turpentine in Hæmoptysis

**Published:** 1860-04

**Authors:** 


*Turpentine in Hæmoptysis.*—There are several well-known remedies which justly enjoy a high reputation for arresting attacks of hæmoptysis, and amongst them may be mentioned acetate of lead, gallic acid, and dilute sulphuric acid. These we see commonly employed, and almost invariably with success. From some cause or other, however, they will sometimes fail, and our reliance must be placed upon some other astringent and styptic, which shall have the power of effectually checking this slow form of bleeding from the lungs. The oil of turpentine is, perhaps, one of the best next to those we have mentioned, and when properly administered can be relied upon. We lately observed two cases of hæmoptysis in the Charing-cross Hospital, under Willshire's care, which continued obstinately persistent, in spite of the free use of acetate of lead firstly, then gallic acid, and thirdly dilute sulphuric acid. One patient was a young man aged twenty-one years, who has had several recurring attacks of this symptom; he was admitted on the 28th of November. The hæmorrhage was stopped only when the oil of turpentine was administered in doses of twenty-five drops three times a day in a little syrup-and.water.

The other patient was a female, at first in the surgical wards under Mr. Hancock's care; she had had a breast amputated, which was followed by intense congestion of the lungs, with hæmorrhage. She was now transferred to Dr. Willshire's care, and after taking the other remedies in full doses without effect, the turpentine completely controlled the bleeding, and she is gradually improving.

The efficacy of turpentine is well-known in hæmorrhages from the urinary passages, and also from the uterus,—that is to say in their passive form; and as it exerts a specific and peculiar influence upon mucous surfaces generally, we may look for good results in other parts of the body, of which the bronchi are most certainly not the least important.—*London Lancet.*

